# Testis-Sparing Surgery for Non-Palpable Leydig Cell Tumors in Prepubertal Children

**DOI:** 10.3390/pediatric12030020

**Published:** 2020-10-26

**Authors:** Vassilis Lambropoulos, Antonios Theodorakopoulos, Vasileios Mouravas, Elissavet Pazarli, Dimitrios Godosis, Chrysostomos Kepertis, Kleanthis Anastasiadis, Ioannis Spyridakis

**Affiliations:** 1 2nd Paediatric Surgery Department, Aristotle University of Thessaloniki, “Papageorgiou” General Hospital, 56403 Thessaloniki, Greece; vmouravas1@gmail.com (V.M.); konpal1453@yahoo.gr (D.G.); ckepertis@gmail.com (C.K.); kaanastasiadis1@gmail.com (K.A.); ispyrida@auth.gr (I.S.); 2 Radiology Department, “Papageorgiou” General Hospital, 56403 Thessaloniki, Greece; atheodorakopoulos@gmail.com; 3 Pathology Department, “Papageorgiou” General Hospital, 56403 Thessaloniki, Greece; elsapazarli@gmail.com

**Keywords:** Leydig cell tumor, testis, children, organ sparing surgery

## Abstract

Leydig cell tumor (LCT) is an infrequent stromal neoplasm of the testis with an incidence of less than 3% of all gonadal tumors in the general male population. Only 25% is found in prepubertal children, where Leydig cell tumors are always reported benign. The hospital records of two prepubertal male children, who underwent organ sparing surgery for testicular LCT the last five years, were retrospectively reviewed. In both of them, the lesion was incidentally found during a scrotal ultrasonography for testicular pain. The diagnosis of a benign LCT was based on the pre-operative physical examination and imaging (Ultrasound-US, Magnetic Resonance Imaging-MRI) as well as the negative tumor markers. A testicle-sparing procedure was decided and the pathologic examination of the surgical specimen confirmed the diagnosis. No tumor recurrence was noted on follow-up. Testis-sparing surgery provides the possibility of complete excision of such lesions and should be considered as the treatment of choice.

## 1. Introduction

Leydig cell tumor (LCT) is an infrequent stromal neoplasm of the testis with an incidence of less than 3% of all gonadal tumors in the general male population [1,2]. In children of prepubertal age, the incidence increases up to 9% of all primary testicular masses [3]. In the adult population, 10% exhibits malignant behavior in contrast to the childhood where Leydig cell tumors are always reported benign [4,5,6]. Usually it occurs in adulthood and only 25% is found in prepubertal children [7]. In most cases, it is an incidental finding during a scrotal sonographic evaluation for other reasons, since in childhood is usually presented as a small sized, non-palpable intratesticular lesion. If hormonally active, they secrete a variety of hormones which may give an onset of endocrine signs, usually before the development of a palpable mass [8]. On scrotal ultrasound (US), these lesions appear as hypoechogenic masses [9]. Contrast-enhanced Magnetic Resonance Imaging (MRI) is essential for the differential diagnosis [10]. Tumor markers such as alpha-fetoprotein (AFP), beta human chorionic gonadotropin (bhCG) and lactate dehydrogenase (LDH) are usually within normal values, in small non palpable lesions in children. Testis-sparing surgery should be considered as the treatment of choice for such cases [6].

## 2. Case Reports

**Figure 1:**
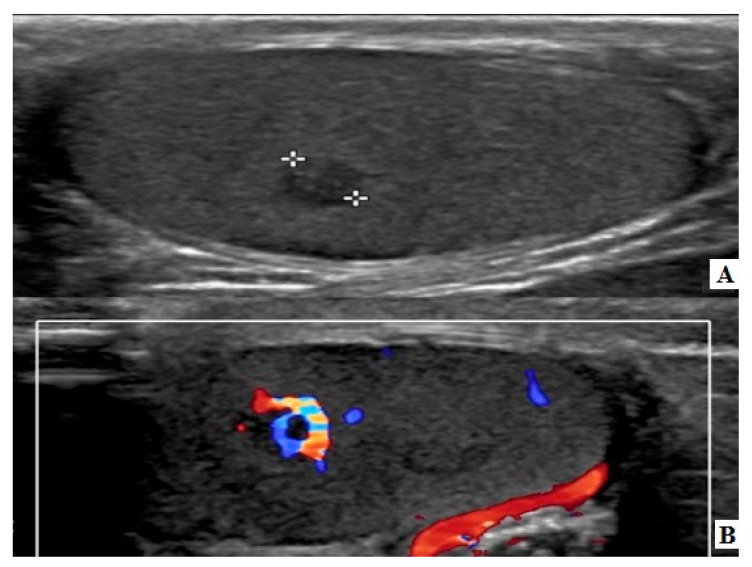
Ultrasound (US) images, in B (**A**) and color Doppler mode (**B**), depicting a hypoechoic lesion with intense vascularization due to a prominent peripheral arterial vessel.

**Figure 2:**
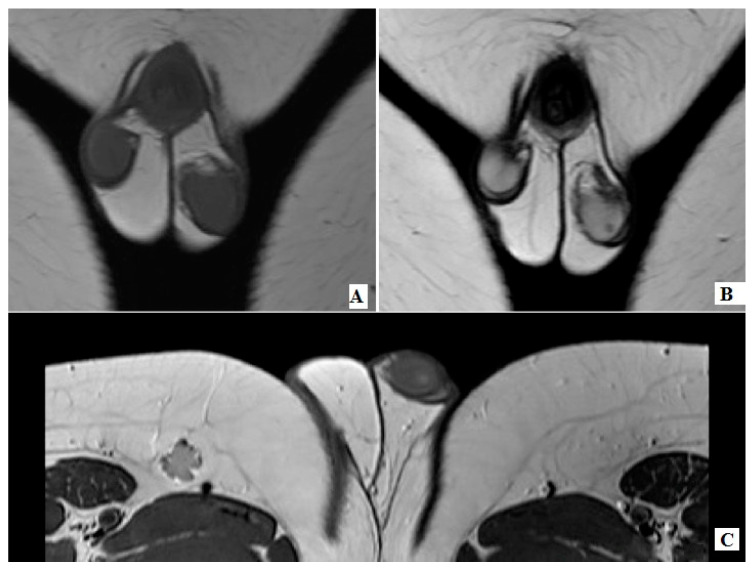
(**A**) Magnetic resonance imaging (MRI) depicting the lesion as isointense on T1-WI, (**B**) hypointense on T2-WI, (**C**) and with intense enhancement after administration of endovenous contrast material.

The hospital records of two prepubertal male children who underwent organ sparing surgery for testicular LCT the last five years were retrospectively reviewed. Their age was 10 and 11 years old, respectively, and both of them underwent a scrotal US exam due to testicular pain, which could not be related to any pathology on clinical examination, as the first child was complaining about pain on the ipsilateral side of the lesion and the latter to the contralateral side. Physical examination of the external genitalia in both patients revealed normal testis in position, size and consistency. No palpable masses were noted. The testicular sonographic findings revealed a hypoechoic mass with a diameter of 3 and 3.4 mm, respectively. In both cases, there was a clear demarcation from the surrounding normal testicular tissue, and on Color Doppler evaluation they both presented a rim of marginal blood flow. No intratesticular calcifications were noted (Figure 1). Due to the nodule’s small dimensions, the position coordinates of the lesion were determined in three axes, transverse, sagittal and coronal so that the surgeon could have a dissection plane corresponding to the US findings. Our patients underwent a scrotal MRI in order to evaluate the intratesticular mass and contribute to the surgical treatment. The lesions were well circumscribed isointense on T1-WI, hypointense on T2-W1 and demonstrated intense enhancement after intravenous administration of contrast material (Figure 2). A preoperative diagnostic orientation towards LCT was made. Both males were evaluated by a pediatric endocrinologist and no hormonal or developmental abnormalities were found. Tumor markers were within normal range. No intra-abdominal or inguinal lymph nodes were found on US exam and chest X-rays were normal. The surgeon’s choice of testicle sparing surgery had the parents’ consent in both cases.

**Figure 3:**
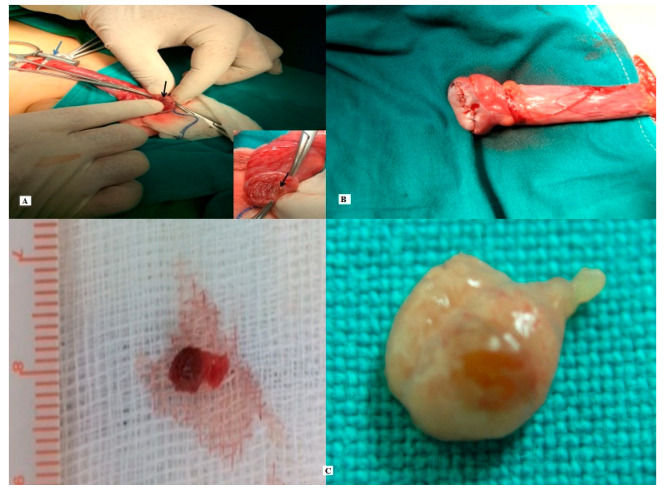
(**A**) Vascular clamp (blue arrow), Leydig cell tumor (LCT, black arrow). (**B**) Testis reconstruction, (**C**) Surgical specimens.

**Figure 4:**
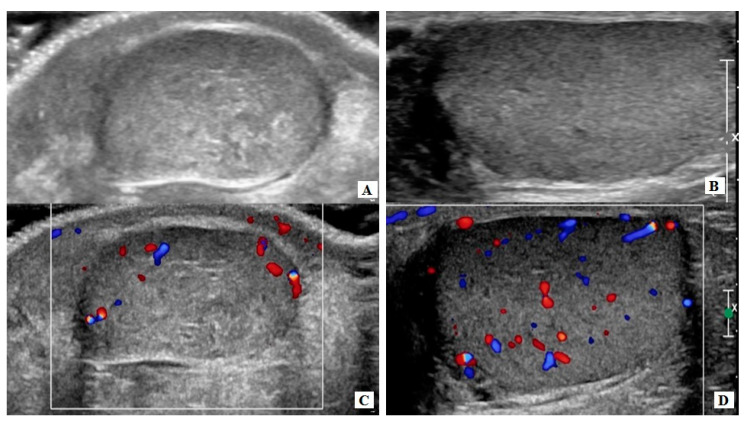
(**A**,**B**) No evidence of the lesion in B-mode and in color Doppler mode, immediately after surgery, (**C**,**D**) and in late follow up.

Through a standard inguinal incision, the spermatic cord was isolated and clamped at the level of the internal inguinal ring, using a soft vascular clamp. The affected testis was then delivered into the wound and placed into a separate operative field in order to avoid tumor spillage. The gubernaculum testis was sectioned, and the tunica albuginea was opened longitudinally, having in mind a dissection plane corresponding to the US findings. The lesion was easily recognized due to its characteristic macroscopic appearance as a yellow to soft brown mass, well demarcated from the surrounding normal testicular tissue. The nodule as well as a 2–5 mm rim of normal tissue around were excised and sent for pathologic examination. The tunica albuginea was repaired using a 6.0 absorbable running suture and the vascular clamp was removed (Figure 3). The ischemia time was recorded and did not exceed 30 min (28 and 22 min, respectively). The organ was re-inserted into the scrotum. On discharge, both patients were evaluated with a US of the testis which confirmed the complete excision of the lesion (Figure 4).

**Figure 5:**
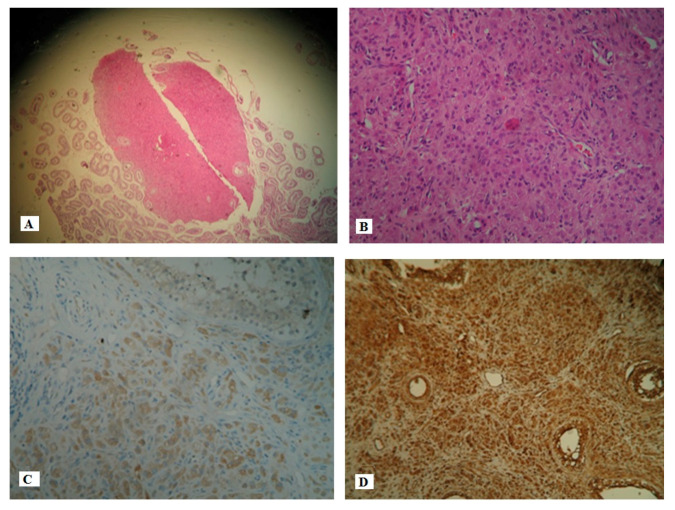
(**A**) Hematoxylin–eosin (H/E), magnification 10×. Well circumscribed nodule with solid architecture. (**B**) Hematoxylin–eosin (H/E), magnification 200×. Neoplasm characterized by solid growth of polygonal cells with eosinophilic granular cytoplasm. No evidence of mitosis, nuclear atypia and/or necrosis. (**C**) Magnification 200×. Neoplastic cells are positive for a-inhibin immunostain. (**D**) Magnification 200×. Neoplastic cells are positive for vimentin immunostain.

The pathologic examination of the surgical specimens confirmed the pre-operative diagnosis. Macroscopically the tumor was well- circumscribed, within the testicle. Microscopically, the tumor was composed of large polygonal cells with eosinophilic glanular cytoplasm and round nuclei. The histological diagnosis of benign LCT was made based on the typical morphological characteristics of the tumor cells (proposed by Kim et al.) [5] and included the small size of the tumor (~3 mm), the intratesticular localization of the tumor with non- infiltrating margins, the absence of atypia, necrosis and/or angioinvasion, and the low mitotic index. Immunohistochemically, the tumor cells were positive for inhibin-α and vimentin (Figure 5).

The follow-up of these patients—24 and 84 months post-op, respectively—with clinical examination, ultrasonography (Figure 4) and measurement of tumor markers, as well as endocrinological evaluation, revealed no LCT relapse and normal testicular growth.

## 3. Discussion

The interstitial cells of Leydig are situated adjacent to the seminiferous tubules in the testicle and produce testosterone under the influence of luteinizing hormone (LH) [8]. Testicular tumors comprise only 1% of solid tumors during childhood and LCTs are even rarer, since they present only 4–9% of all primary testicular tumors in this age group [3,11,12]. The affected boys usually present with isosexual precocious pseudopuberty due to the heightened secretion of androgens, mostly testosterone. Even in these symptomatic cases the course of LCTs is considered benign in childhood [4,5,6]. The amplified use of scrotal US has contributed to the early detection of non-palpable indolent masses in asymptomatic prepubertal boys.

Sonographic findings of the affected testicle usually demonstrate a small hypoechoic nodule, well defined, relatively homogeneous, which can contain faint internal echoes corresponding to small areas of hemorrhage or fibrosis, with intense peripheral rim of vascularization due to a hypertrophic arterial vessel originating from a capsular artery [13,14,15]. US sensitivity and specificity of the examined lesions is 96% and 44%, respectively [15]. The pre-operative differential diagnosis can be clarified by the use of contrast-enhanced MRI [10,13]. Its major disadvantages are the high cost and the need for sedation. A US examination could be the only diagnostic tool. However, due to the low incidence of these tumors in every single institution, surgeons usually demand a complete preoperative diagnostic approach, especially for uncertain cases. Characteristic findings include iso- or intermediate signal intensity on T1-WI, low signal intensity in T2-WI, intense and homogeneous enhancement after intravenous administration of contrast.

Tumor markers AFP, bhCG and LDH, should always be measured not only in order to exclude malignancy, but also for the post-operative follow-up of the patients. In case of hormone-active lesions, an endocrinological evaluation should be carried out in order to exclude other hormone secreting diseases that may cause secondary Leydig cell hyperplasia.

Since we had to treat asymptomatic patients with unilateral, incidentally found on US examination testicular LCT lesion, which was non-palpable, testicle-sparing surgery (TSS) was the best option. It provides the possibility of complete excision of the lesion and has no impact on the child’s psychological encumbrance, since there are good functional and cosmetic results. We consider radical orchidectomy as an overtreatment. Parents should be aware of the possibility of synchronous or metachronous bilaterality of these lesions and give their consent on TSS [14]. Intra-operative use of US, which is unfortunately not available in our hospital, could probably further reduce the ischemia time which should always be kept less than 30 min duration. The surgical specimen was not sent for frozen section analysis due to the pre-operative favorable physical examination and imaging (US, MRI), the negative tumor markers, the very small lesion’s size (less than 4 mm) and the characteristic macroscopic intra-operative image of the nodule.

The permanent histology for benign LCT is based on the criteria suggested by Kim et al., which include the dimensions of the nodule, the margins of the mass (whether or not free), the extra-testicular extension, the presence of angioinvasion or necrosis, atypias and the high mitotic index [5]. These criteria are further completed by Cheville et al., including ploidy and proliferation rate [4].

Despite the fact that these lesions have shown a favorable outcome, a follow-up is essential for a long period with physical and imaging (US) examination. Until now, there is only one case of malignant LCT in a 9-year-old child, but with bilateral testicular involvement and with mass dimensions on one side greater than 6 cm [16].

A multidisciplinary approach including pediatric surgeons, endocrinologists, radiologists and pathologists leads to a precise diagnosis which is essential for choosing the right way of surgical treatment.

Testis-sparing surgery should always be performed in such benign cases. There is no impact on the child’s psychological encumbrance since there are good functional and cosmetic results, and the prepubertal patient has a better chance of fathering children.
